# Evaluating Provider Advice and Women’s Beliefs on Total Weight Gain During Pregnancy

**DOI:** 10.1007/s10903-015-0162-8

**Published:** 2015-02-04

**Authors:** Nkiruka V. Arinze, Sharon M. Karp, Sabina B. Gesell

**Affiliations:** 1 Vanderbilt University School of Medicine, Nashville, TN USA; 2 Schools of Nursing and Medicine (Pediatrics), Vanderbilt University, Nashville, TN USA; 3 Division of Public Health Sciences, Department of Social Sciences and Health Policy, Wake Forest School of Medicine, Maya Angelou Center for Health Equity, Medical Center Boulevard, Winston-Salem, NC 27157 USA

**Keywords:** Gestational weight gain, Hispanic, Obesity, Health disparities

## Abstract

Excessive gestational weight gain (GWG) is associated with complications for both mother and child. Minority women are at increased risk for excessive GWG, yet are underrepresented in published weight control interventions. To inform future interventions, we examined the prevalence and accuracy of provider advice and its association with personal beliefs about necessary maternal weight gain among predominantly Latina pregnant women. Secondary analysis examining baseline data (N = 123) from a healthy lifestyle randomized controlled trial conducted in and urban area of the South East. Only 23.6 % of women reported being told how much weight to gain during pregnancy; although 58.6 % received advice that met Institute of Medicine recommendations. Concordance of mothers’ personal weight gain target with clinical recommendations varied by mothers’ pre-pregnancy weight status [*χ*_(4)_^*2*^ = 9.781, *p* = 0.044]. Findings suggest the need for prenatal providers of low-income, minority women to engage patients in shaping healthy weight gain targets as a precursor to preventing excessive GWG and its complications.

## Introduction

In 2009, the Institute of Medicine (IOM) revised its guidelines for appropriate weight gain during pregnancy, taking into account the growing prevalence of obesity in women of childbearing age [1]. (Table 1) Over 40 % of pregnant women exceed the IOM recommendations for gestational weight gain (GWG), with overweight and obese women at increased risk [2]. Excessive weight gain during pregnancy is associated with complications in delivery [3], increased postpartum weight retention [4], persistent obesity [4], increased BMI in offspring in childhood, adolescence and adulthood [5], and longer maternal hospital stay after labor and delivery [5].

**Table 1:** Clinical recommendations for cumulative gestational weight gain by pre-pregnancy BMI for mothers of singletons and twins at gestational ages 37–42 weeks (IOM, 2009**)**

Prepregnancy BMI	Recommended range of Cumulative weight gain (lbs)	
	Singleton	Twins
Underweight (<18.5 kg/m^2^)	28–40	–
Normal (18.5–24.9 kg/m^2^)	25–35	37–54
Overweight (25.0–29.9 kg/m^2^)	15–25	31–50
Obese (≥30.0 kg/m^2^)	11–20	24–42

Actual weight gain during pregnancy is strongly influenced by provider recommendation [6, 7]. Evidence shows that women’s actual weight gain during pregnancy is strongly influenced by personal beliefs and healthcare providers’ advice [6, 7]. When women receive weight gain advice that is inconsistent with IOM recommendations (i.e., above or below), they are 3.6 times more likely to gain more or less than IOM recommendations, respectively [7]. Women whose personal weight gain targets are below IOM recommendations are five times more likely to gain less than recommended, and women with targets above the recommendations are six times more likely to gain more than recommended [7].

There is limited data about the beliefs of Latinas on GWG even though the problem of excessive weight gain during pregnancy is particularly important for this group which now comprises 20 % of new mothers in the US [1]. At 65.4 %, the prevalence for overweight and obesity in Latinas aged 20–39 years is higher than the national prevalence for all women in that age group [8]. This increased rate of overweight and obesity puts Latinas at increased risk for excessive GWG. The purpose of this paper is to examine the prevalence and accuracy of provider advice on maternal weight gain and its association with personal beliefs about necessary maternal weight gain within a sample of predominantly Latina pregnant women who received prenatal care in their dominant language.

## Methods

### Study Design and Participants

We examined baseline data (N = 123) from a randomized controlled trial, Madre Sana, Bebé Sano (Healthy Mother, Healthy Baby), designed to prevent excessive GWG. IRB approval was obtained from Vanderbilt and Wake Forest Universities.

#### Eligibility Criteria

Eligibility criteria included Spanish or English speaking women, ≥16 years, 10–28 weeks gestation, receiving prenatal care, planning to stay in the area for the full pregnancy, and willing to authorize release of medical information for the purpose of data abstraction.

#### Recruitment

Recruitment and enrollment took place at various obstetric clinics in an urban area of the South East from January–April 2011.

#### Consent/Assent Process

Eligible women were informed of the study and, if interested, consented in their language of preference (Spanish/English).

### Measures

The study questionnaire was administered by trained study personnel in Spanish or English in participants’ homes.

#### Pre-Pregnancy Body Mass Index (BMI)

Self-reported pre-pregnancy weight and measured height collected at baseline were used to calculate pre-pregnancy BMI [9].

#### Provider Advice on Gestational Weight Gain and Weight Gain Target

Women were asked (1) “Has your health professional told you how many pounds (kilos) you should gain during this pregnancy?” (2) “How many pounds (kilos) did your health professional say you should gain from the beginning to the end of this pregnancy?” and (3) “How many pounds (kilos) do you think you should gain from the beginning to the end of this pregnancy?” Responses were compared to the 2009 IOM recommendations.

#### Sociodemographics

The demographic variables used in this study included maternal age, country of origin, ethnicity, education, language preference, years spent in the US, parity, use of tobacco, alcohol and prenatal vitamins during pregnancy, employment, and participation in the Special Supplemental Nutrition Program for Women, Infants and Children (WIC).

### Statistical Analysis

Chi- squared tests were used to compare frequency distributions. *P* < 0.05 was considered significant.

## Results

### Sample Demographics

The mean age of respondents (N = 123) was 26.3 years (SD 5.9). Women classified themselves as Latina (78 %), African–American (14 %), Caucasian (4 %), or other (4 %). Approximately three-quarters of the women were immigrants (75 %), primarily from Mexico and Honduras. All women received prenatal care in their dominant language (Spanish/English) (Table 2 **).**

**Table 2:** Sample demographics (N = 123)

	N (%)
*Maternal age (years)*	
<18	4 (2.8)
18–24	60 (41.7)
25–30	42 (29.2)
>30	38 (26.4)
*Pre*-*pregnancy BMI*	
Under	0 (0)
Normal	48 (39.0)
Overweight	41 (33.3)
Obese	34 (27.6)
*Country of origin*	
United States	30 (24.4)
Mexico	59 (48.0)
Honduras	13 (10.6)
Other Latin Am. country	16 (13.0)
African country	3 (2.4)
Other country	2 (1.6)
*Highest education level*	
Eighth grade or below	26 (21.1)
Some HS, no diploma	51 (41.5)
HS diploma/GED	28 (22.7)
Some college, vocational/technical degree	13 (10.6)
Bachelor’s degree or higher	5 (4.1)
*Language of preference*	
English	29 (23.6)
Spanish	85 (69.1)
English and Spanish	9 (7.3)
*WIC*	
No	48 (39.3)
Yes	74 (60.7)
*Expecting Twins*	
No	121 (98.4)
Yes	2 (1.6)
*Previous pregnancies*	
0	42 (34.1)
1	34 (27.6)
2	24 (19.5)
3	16 (13.0)
4	4 (3.3)
5	2 (1.6)
6	0 (0)
7	1 (0.8)
*Smoked after pregnancy was confirmed*	
No	110 (90.2)
Yes	12 (9.8)
*Consumed alcohol after pregnancy was confirmed*	
No	111 (91.0)
Yes	11 (9)
*Taking prenatal vitamins*	
No	9 (7.3)
Yes	114 (92.7)
*Currently employed*	
No, by choice	17 (13.9)
No, not by choice	56 (45.9)
Yes, but with an irregular schedule	10 (8.2)
Yes, part time	18 (14.8)
Yes, full time	21 (17.2)

### Provider Advice on Gestational Weight Gain

Although all of the women had at least one prenatal appointment before completing the baseline survey, only 24 % reported that their healthcare provider told them how much weight to gain during their pregnancy. Receiving advice did not vary by pre-pregnancy BMI [*χ*_(2)_^*2*^ = 0.421, *p* = 0.810].

Of those who reported receiving advice on GWG, 59 % (17/29) reported receiving advice that fell within and 31 % (9/29) that was above the IOM recommendations, and 10 % (3/29) could not remember the advice. The concordance of providers’ weight gain advice with IOM recommendations did not vary by mothers’ pre-pregnancy BMI [*χ*_(2)_^*2*^ = 2.382, *p* = 0.304].

### Personal Gestational Weight Gain Target

When asked how much weight they thought they should gain during their pregnancy, 25 % of participants had personal weight gain targets below the IOM target range 49 % had targets within the recommended range 15 % had weight gain targets above the recommended range and 11 % did not know how much weight to gain. The concordance of mothers’ personal weight gain target with IOM recommendations varied by their pre-pregnancy weight status: Normal weight mothers were more likely to have low weight gain targets, whereas overweight and obese women were more likely to have high weight gain targets (Table 3).

**Table 3:** Association of mother’s personal gestational weight gain targets and their pre-pregnancy weight category

		Pre-pregnancy BMI			
		Normal	Overweight	Obese	Total
Accuracy of mother’s weight gain target	Under IOM rec	18 (40.9 %)	6 (16.7 %)	7 (23.26 %)	31 (28.2 %)
	Within IOM rec	23 (52.3 %)	23 (63.9 %)	15 (50 %)	61 (55.5 %)
	Above IOM rec	3 (6.8 %)	7 (19.4 %)	8 (26.7 %)	18 (16.4 %)
		44 (100 %)	36 (100 %)	30 (100 %)	110 (100 %)

Only 110 of the 123 women in the sample responded to the survey question about personal weight gain target

*χ* ^2^(4) = 9.781, *p* = 0.044

The concordance of mothers’ weight gain targets with IOM recommendations also varied by receipt of professional advice. Those receiving advice were more likely to have accurate personal weight gain targets, whereas those women who did not receive professional advice were more likely to have low or high personal weight gain targets (Table 4).The concordance of mothers’ weight gain targets with IOM recommendations did not vary by language preference, years in the US, maternal education, or WIC status.

**Table 4:** Association of mother’s personal gestational weight gain targets and their provider giving advice on appropriate weight gain

		Professional advice on weight gain received		
		No	Yes	Total
Accuracy of mother’s weight gain target	Under IOM rec	28 (34.1 %)	3 (11.1 %)	31 (28.4 %)
	Within IOM rec	40 (48.8 %)	21 (77.8 %)	61 (56.0 %)
	Above IOM rec	15 (18.1 %)	3 (11.1 %)	17 (15.6 %)
		83 (100 %)	27 (100 %)	110 (100 %)

*χ* ^2^(2) = 7.519, *p* = 0.023

## Discussion

Despite the attention garnered by the 2009 revision of the IOM’s guidelines for weight gain during pregnancy, many of the low-income, minority women in this sample did not receive information about appropriate GWG. This vulnerable population is already at increased risk of excessive GWG and the concomitant adverse effects on mother and child.

Provider advice is key in helping women meet appropriate GWG targets [6]. While less than a quarter of women in our study reported receiving any information on weight gain, those who did were more likely to have target weights within clinical recommendations. Furthermore, most participants who reported receiving advice on GWG got recommendations within the current IOM standards.

Within our sample, provider advice and accuracy did not vary based on women’s pre-pregnancy BMI. Yet similar to other reports in predominantly white women [6], we found that pre-pregnancy BMI is strongly associated with Latina women’s target weight gain. Together, these findings suggest that providers are as comfortable discussing weight gain restrictions with overweight and obese women as they are with normal weight women, but that overweight and obese women still have weight gain targets in excess of clinical guidelines. Providers may need to re-emphasize their recommendations with overweight and obese patients to help align their personal weight gain targets with current clinical recommendations.

In the few women in our sample (<25 %) who reported receiving provider advice on appropriate weight gain, 31 % received recommendations in excess of current clinical guidelines. Both the lack of receipt of clinical guidance and the recollection of inaccurate information observed in this study are of concern because receipt of provider advice has been shown in prior work to be the most important predictor of a woman’s weight gain target [6].

Women who have a weight gain target that is concordant with IOM recommendation are less likely to have excessive weight gain [4]. In our study, normal weight mothers were more likely to have low weight gain targets, whereas overweight and obese women were more likely to have high weight gain targets. This is of concern because excessive weight gain is associated with postpartum weight retention and persistent maternal obesity [4].

Accuracy of women’s weight gain target did not vary by acculturation (language preference, years in the US). Our study compared women who had been in the US for fewer or more than 5 years. Other work has shown that generational acculturation was positively associated with excessive GWG [10]. While low maternal education has been associated with higher rates of excessive weight gain [2], our study did not find any significant effect of maternal education on weight gain target.

Understanding beliefs about appropriate weight gain during pregnancy is crucial for intervention planning. The strength of this study is its focus on minority women who are underrepresented in published GWG interventions yet carry increased burden of gaining excessively during pregnancy. Three-quarters of participants reported not receiving advice on appropriate weight gain during early prenatal care visits. However, those who did receive advice were more likely to have personal weight gain targets in line with current clinical recommendations. These data suggest that prenatal care providers of low-income, minority women in particular should make healthy maternal weight gain targets a priority in their care to prevent excessive weight gain during pregnancy and the associated complications.
